# A245 A TERTIARY CENTRE REVIEW OF PORTO-SYSTEMIC SHUNTS: AN INCREASINGLY IMPORTANT TOOL IN LIVER DISEASE MANAGEMENT

**DOI:** 10.1093/jcag/gwac036.245

**Published:** 2023-03-07

**Authors:** S Malik, G Hirschfield, A Jaberi

**Affiliations:** 1 Faculty of Medicine, University of Toronto; 2 Toronto Center for Liver Disease, University Health Network; 3 Vascular Interventional Radiology, Joint Department of Medical Imaging, Toronto, Canada

## Abstract

**Background:**

Portal hypertensive complications arising from chronic liver disease are increasingly prevalent. Radiologically placed porto-systemic shunts are used successfully to address complications such as ascites and recurrent variceal bleeding.

**Purpose:**

We sought, with approval from ethics to electronically chart review the UHN experience for TIPSS (transjugular intrahepatic porto-systemic shunt) and DIPS (direct intrahepatic porto-caval shunt) over time.

**Method:**

We employed a search strategy focused on radiologic databases, to identify UHN patients who had received a porto-systemic shunt insertion over three decades between January 1, 1990, to September 14, 2021. A total of 238 UHN patients that had a shunt imaged, re-assessed, or inserted were included in this retrospective electronic chart review. Demographic information was recorded at baseline. Data pertaining to clinical outcomes of portal hypertension such as refractory ascites, recurrent gastrointestinal bleeding and ESLD progression was recorded at baseline, 1 month, 3 months and 6 months post-shunt insertion. HVPG was recorded pre-shunt and post-shunt insertion. Transplant-free survival and post-operative complications was also assessed. Patients were followed until death or liver transplant.

**Result(s):**

Out of 238 patients confirmed, 219 had a TIPSS insertion and 19 a DIPS insertion. The average age at time of shunt was 55.1 ± 8.4 years. 53.7% (n=128) of patients were male. Indications for portosystemic shunt placement included refractory ascites (n=141), recurrent gastrointestinal bleeding (n=73), hydrothorax (n=10), hepatorenal syndrome (n=8), and portal vein thrombosis (n=4). Etiology of disease was categorized as alcohol-associated liver disease (n=107), non-alcohol associated fatty liver disease (n=36), viral hepatitis (n=27), autoimmune hepatitis (n=38) and vascular liver disease (n=20). Relevant events noted post procedure included hepatic encephalopathy (n=34), hydrothorax (n=17), vascular (including stent) thrombosis (n=17) and renal failure (n=4). 12 months post-shunt insertion 2.5% (n=6) of patients had died or received liver transplant; of these 1.7% (n=4) were transplanted. HPVG fell consistently post-procedure: 15.6 ± 1.5mmHg pre-shunt to 5.9 ± 1.2mmHg post-shunt. There was no observable increase in MELD-Na score in the first month post-shunt. Temporal trends showed that more patients aged 50 to 69, and more female patients, are now receiving shunt insertion than at the initiation of the chart review.

**Image:**

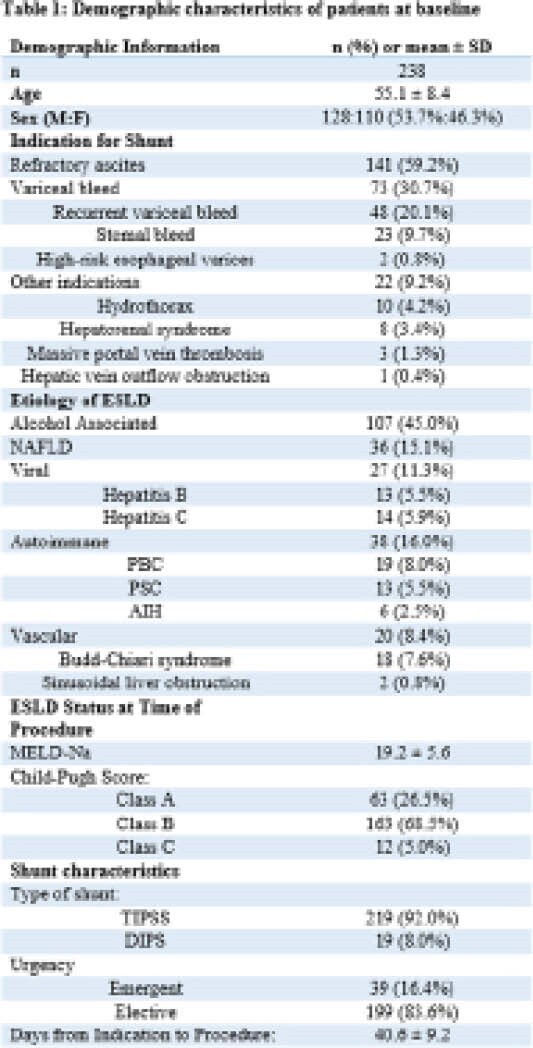

**Conclusion(s):**

Portosystemic shunt placement in the form of TIPSS or DIPS insertion is an increasingly common and effective treatment for portal hypertension in patents with advanced liver disease at UHN.

**Disclosure of Interest:**

None Declared

